# Peer assumption: an illusory consensus hidden in the criminal responsibility of juvenile offender—evidence from psychology

**DOI:** 10.3389/fpsyg.2024.1321870

**Published:** 2024-05-09

**Authors:** Sanyang Liu, Yangxue Su, Yumiao Fu, Haifeng Li, Dayong Xu, Min Zhou, Weibo Jian

**Affiliations:** ^1^Kaiyuan Law School, Shanghai Jiao Tong University, Shanghai, China; ^2^Law School, Law School of Jiangsu Normal University, Xuzhou, China; ^3^Legal Education and Research Center for Minors, Jiangsu Normal University, Xuzhou, China; ^4^Institute of Criminal Law, Jiangsu Normal University, Xuzhou, China; ^5^Shandong Mental Health Center, Ji’nan, China; ^6^The First Branch of Zhangdian Tax Bureau, Zibo, China; ^7^Xuzhou People's Procuratorate, Xuzhou, China; ^8^Huai 'an People's Procuratorate, Huai'an, China; ^9^School of Law and Economics, Sapienza University of Rome, Rome, Italy

**Keywords:** juvenile delinquent, criminal responsibility, dialectical thinking, self-control, empathy

## Abstract

**Introduction:**

There is a consensus hidden in the criminal legislation of many countries that the criminal responsibility capacity of juvenile offenders is not significantly different from that of their peers. The purpose of this paper was to test this hypothesis. The research objects of this paper were 187 juvenile offenders in J Province, China, who are under detention measures, and 2,449 students from junior high school, senior high school and university in S Province as comparison objects. We subjected the gathered materials to independent-samples *t*-tests and one-way analysis of variance (ANOVA).

**Results:**

(1) The self-control ability (109.30, 123.59) and empathy ability (63.86, 72.45) of juvenile offenders were significantly different from those of ordinary minors, but the difference of dialectical thinking ability was not statistically significant; (2) Except for the influence of mother’s education level and family income on dialectical thinking ability, the other variables had no statistical significance on the three kinds of ability. Therefore, it was suggested that the correction plan and means for juvenile offenders should focus on the improvement of self-control ability and empathy ability.

## Introduction

In 2006, a book named “Shocking Call - Yulin Juvenile Crime Warning Record” was published in Shaanxi, China, although it did not attract much attention at the beginning of publication. However, decades later, this book became one of her representative works. This book recorded a series of disturbing and frightening phenomenons of juvenile delinquency in northern Shaanxi at that time that teenagers, ranging in age from 13 to 22, committed murder, rape and robbery in groups of two or three in Yulin, Shaanxi, motivated by so-called “friendship,” “self-realization” or simply “courage exercise.” Almost all of the perpetrators, who were insensitive at the time of their actions, appeared extremely depressed and remorseful when faced with trial, many of them even refusing to seek a defense (Gao and Gao, 2006). On the one hand, from the perspective of culpability theory, criminal condemnation of juvenile offenders is ethically justified because of the more or less cognition or such possibility of most behaviors that violate basic moral culture (Loewy, 1987; Arenella, 1991; Smith, 2009). On the other hand, because of functions of brain still in the proven stage of growth, compared with adults, “adolescents as a class operate under a comparatively reduced capacity when it comes to higher executive function, including autonomous choice, risk perception, self-management, and calculation and comprehension of future consequences” (Carroll, 2015). Thus, it is generally not suggested to impose exactly the equal penalty on a minor as an adult in the same circumstance, such as death penalty (American Psychological Association, 2004). In addition, research from the psychological and neurological literature suggests that because of the transient nature of this stage of development, dmany of the irrational behaviors that adolescents exhibit during adolescence do not usually carry over into adulthood (Scott et al., 2016). Therefore, their criminal treatment is necessarily different from that of persons born with intellectual disabilities (Carroll, 2015). Its no doubt, therefore, that “Few issues challenge a society’s ideas about both the nature of human development and the nature of justice as much as serious juvenile crime” (Steinberg, 2017).

Although it is recognized that the psychological state of adolescents is different from that of adults, national legislation reflects different positions on whether this difference should be recognized at the level of criminal responsibility. The first type of criminal legislation is the criminal legislation that takes discrimination as the basis for the perpetrator to bear criminal responsibility, such as France (Zhu, 2016) and Switzerland (Xu, 1998). The second type of criminal legislation based on age as the basis of criminal responsibility can be divided into several cases. (1) Countries that adopt dichotomous age of criminal responsibility legislation, such as Spain adopting the age standard of 18 years old (Pan, 2004), the United States adopting the age standard of 16 years old in the Model Criminal Code (Liu and Wang, 2005), Norway adopting the age standard of 15 years old (Ma, 2005), and Germany (He and Lin, 2017), South Korea (Kim, 1996), Italy (Huang, 2007), and Japan (Chen, 2016) adopted the age standard of 14 years. (2) Countries that adopt a trichotomous age of criminal responsibility legislation, such as Albania. It sets 12 to 14 years of age as the restricted age of criminal responsibility, bearing criminal responsibility for specific types of crime, and set the age of full criminal responsibility above 14 years old and assume criminal responsibility for all crimes (Compilation Office of The General Office of the Standing Committee of the National People's Congress, 1956). What’s more, the criminal legislation of Bulgaria establishes the age of limited criminal responsibility between 14 and 18 years old, and the age of full criminal responsibility above 18 years old (Institute of Law of Chinese Academy of Sciences, 1963). (3) Countries that adopt the age of criminal responsibility legislation with multiple methods divide the age of criminal responsibility of juvenile offenders into several stages. At a lower age stage, juvenile offenders bear criminal responsibility only for specific types of crimes. And at a higher age stage, they bear criminal responsibility for all crimes, but enjoy the legal preference of mitigating punishment. The criminal codes of Russia (Huang D, et al., 1996), Poland (Chen Z, 2009), Thailand (Wu G, 2004) and Chinese Mainland (Criminal Law, 2019) all adopt this legislative model. The third type of criminal legislation is the criminal legislation that adopts both age standards and discrimination standards. The criminal legislation of the Netherlands (Yan and Ge, 2008), the United Kingdom, some states in the United States and Hong Kong, China (Tan and Di, 2022) adopts this model, which defines a certain higher age level as the age of full criminal responsibility. And delimit a lower age range to determine the imposition of criminal responsibility according to the discerning power of juvenile offenders. These laws reflect the extensive attention and continuous self-examination on the issue of juvenile criminal responsibility capacity worldwidely.

These laws reflected the extensive attention and continuous reflection of countries around the world on the issue of juvenile criminal responsibility capacity.

Indeed, in order to explore the differences between juveniles and adults on the basis of punishment, jurists in various countries have made a lot of efforts. Recently, it is also commendable to introduce the research results of brain science and neurology into criminal justice (Casey et al., 2008). Furtherly, not only is there a lot of consensus among neuroscientists, but this consensus has indeed begun to have an impact on judicial determination. Unfortunately, however, such efforts remain inadequate. As some scholars have realized, at the legislative level, the legislation of all countries mainly focuses on the criminal responsibility of adults, except for the criminal responsibility of juveniles. Under established logical assumptions, human beings always use the responsibility capacity of adults as a frame of reference to conceive the differences that juveniles may have (Carroll, 2015). In the judicial field, practitioners may first conceive the possible differences between ordinary juveniles (juveniles who are not ready to commit or have committed crimes) and ordinary adults (adults who are not ready to commit or have committed crimes), and then determine whether to reduce the responsibility and exempt the criminal responsibility of the criminal juveniles. In other words, we equate ordinary juveniles with criminal juveniles, ordinary adults with criminal adults, punishing or even reforming juvenile offenders according to this plausible materialistic assumption. The introduction of neurological results is based on this logical assumption, and our normative construction of juvenile criminal responsibility capacity is also based on this assumption.

However, this hypothesis may be specious. First of all, if we are sure that A and B have some differences worthy of criminal law’s attention, why can we draw the conclusion that Class A subject should be treated criminally leniently based on the reference to their own circumstances?

Certainly, “Justice is to give everyone he deserves part of the firm and enduring desire,” the normative requirements for juveniles must not be as strict as those for adults, yet, simultaneously, neither can the normative requirements for juveniles be taken as a reference with that for adults.

Secondly, there might also be the deviation in perception if we simply take the research results of brain science as the basis for the identification of criminal responsibility capacity. If the standard of brain health is taken as the standard to measure criminal responsibility and impose penalty simply, illegal people who have violent epilepsy due to brain damage are not responsible and cannot be evaluated according to the standard evaluation standards of ordinary people (Moir and Jessel, 1999). Similarly, criminal of conviction who have long been dominated by external thoughts cannot be measured entirely by the standards of ordinary people, because their mental state does not always seem to be in a normal state neither (Jakobs, 1997). However, no matter how controversial the academic circles of various countries are, these viewpoints have not been stipulated in criminal legislation as a consensus. The capability of responsibility for juvenile offenders can not be generalized, because their psychology is always in a very unstable state. Thus, every time the scope of identification of brain science and neurology is advanced, the evaluation of responsibility capacity in criminal law can only take a step back, and normative evaluation is “shivering” under the shadow of determinism (Ryuichi, 2015). Finally, it does not seem to make sense in theory for us to equate the responsible capacity of criminal individuals with that of non-criminal individuals. In the criminal theories of Germany, Japan and other countries or regions, there exists the basic idea of the possibility of expectation. It advocates that a wrongful actor should be reduced or even excluded the situation of criminal condemnation if can hardly freely carry out legal acts (Liszt, 2006; Noriyuki, 2007; Huang, 2009). In the United States and other countries, the defence be claimed that “But everyone does it!” (BEDT) is also popular (Snyder et al., 1983). Although this defense has been rejected in a number of cases, based on the interpretation of the Model Criminal Code Sec. 2.02 (2) (d), when an ordinary person in the context of the perpetrator usually violates the duty of reasonable care created by the specific provisions, he or she cannot at least be held criminally liable for negligence. Therefore, in the adult context, not all offenders have the same capacity to comply with the norms of duty as others, let alone in the context of juveniles.

In short, based on the theoretical of criminal responsibility, when investigating the criminal condemnation of juvenile offenders, we should investigate the potential differences between juvenile offenders and ordinary adolescents, laying a foundation for the discussion of criminal treatment. At present, the criminal legislation of various countries implicitly assumes that although the criminal responsibility capacity of juvenile offenders is relatively low, it is not necessarily compared with their peers. When they fail to resist the temptation of the outside world and carry out deviant behavior, they should be entirely responsible for themselves in the perspective of juvenile. But does this hypothesis be true? To wit:

*H1*: illegal behaviors are irelevant with adolescent psychological development; The identification and control ability of juvenile offenders is not different from that of their peers.

The main purpose of this study was to test the validity of the underlying hypotheses by quantitative methods. According to the basis of juvenile criminal responsibility (capacity for identification and control), the juveniles’ capacity for criminal responsibility is quantified by three psychological indicators. (1) Indicators of ability of dialectical thinking. The development of capacity for dialectical thinking enables adolescents to understand the world objectively, look upon things comprehensively and deal with problems rationally (Inhelder and Piaget, 1958; Nisbett et al., 2001; Cheng, 2009; Boucher, 2011). Studies have found that there is a negative correlation between the degree of development of capacity for dialectical thinking of adolescents and the frequency of crime risk (Crick and Dodge, 1994; Zhang et al., 2011). (2) Indicators of self-control ability. Self-control refers to the ability to suppress inappropriate emotions and behaviors and replace them with appropriate behaviors (Casey, 2015). Low self-control often predicts the emergence of many bad behaviors, especially participation in antisocial and criminal behaviors (Walters, 2016). The immaturity of the prefrontal cortex provides the physiological basis for the lack of self-control in adolescents (Yurgelun-Todd, 2007; Bell and McBride, 2010). Simultane, due to the direct influence from social and environmental factors (Somerville, 2013; Blakemore and Mills, 2014), especially the peers (Guyer et al., 2012), and the indirect influence of dopamine-rich regions in the ventral striatum of the brain (Chein et al., 2011), the development of self-control in adolescents presents a bi-directional feature.

Psychological research has also found that the development of empathy will have an impact on juvenile delinquency (Narvey et al., 2021). Empathy refers to the cognitive ability to experience and understand the emotions of others (Jolliffe and Farrington, 2004). Low empathy is frequently associated with aggressive behavior and crime, and is a psychological characteristic that increases the likelihood of crime, especially violent behavior (Harpur et al., 1988; Jolliffe and Farrington, 2004, 2006a,b); High empathy is associated with reduced violence and aggressive behavior (Broidy et al., 2003). Therefore, in addition to the indicator of dialectical thinking ability (1) and self-control ability (2), this study also regards the indicator of empathy ability (3) as a measure of the level of juvenile criminal responsibility ability. The H1 we were going to demonstrate before can be modified to H2:

*H2*: illegal behaviors are irelevant with adolescent psychological development; Compared with juvenile offenders, ordinary adolescents' dialectical thinking, self-control and empathy ability may not be higher, and their empathy ability development level (or stable level), dialectical thinking and self-control development level is also the same.

### The current study

Juveniles’ criminal responsibility capacity is the core of its criminal condemnability evaluation, but as mentioned above, many countries in the world regard age as the main indicator of a juvenile’s capacity for responsibility. However, this view not only has invalid foundation, unclear connotation and logical circular argumentation in the level of criminal law, but also exists doubts in the level of criminal law practice. In 2022, an empirical study on 3,208 junior high school, senior high school and freshman students in S Province of the People’s Republic of China showed that although age variables have significant effects on dialectical thinking ability, self-control ability and empathy ability, only empathy ability has a trend of strengthening with the increase of age. However, recognition ability and control ability were negatively correlated with age variables (Shang et al., 2022). Therefore, it is impossible to understand the overall development of juvenile ability of responsibility solely through the growth of age. More attention should be paid to the comparative research on the level of criminal responsibility ability between juvenile offenders and ordinary adolescents. The quantitative analysis method was continued in this study. First, on the basis of the above studies, the scores of questionnaires of the dialectical thinking ability, the self-control ability and the empathy ability, which measures the tendency of violent crime, were compared between ordinary adolescents and juvenile offenders. Secondly, under the premise of controlling the demographic variables such as academic achievement, parental occupation and socioeconomic status, the relationship between the three abilities and the age of juvenile offenders is analyzed. Ultimately, this study attempts to address the following questions:

- Do juvenile offenders differ from their peers in their ability to think dialectically, self-control and empathize?
- If so, in what ways is this difference reflected? What are the reasons for the differences? How do they influence their criminal behavior? Can the existing criminal justice system improve their criminal psychology?
- If not, how to trace the actual conditions and causes of juvenile delinquency? What measures should be taken to reform and eliminate the environmental and family factors that stimulate them to commit crimes?
- What aspects should the existing juvenile criminal justice mechanism be improved to meet the realistic needs of juvenile offenders’ reform?

## Method

### Data

The samples selected for the first time in this study were juvenile offenders in several cities in J Province of the People’s Republic of China. J Province is located in the east of China, and its economic status is at the forefront of the country (J Province *per capita* disposable income [PCDIS] = [¥52,674] in 2023, national *per capita* disposable income [PCDIS] = [¥39,218] in 2023). Teenagers in this province can have access to more cutting-edge and advanced educational resources and educational concepts, and deviant teenagers who receive education and management in the province can also feel relatively enlightened and advanced correction concepts. In order to respect the cultural characteristics and regional differences of J Province, this research group selected juvenile delinquents in SZ and ZJ in southern area of J Province, TZ and NT in midland of J Province, and XZ, HA and SY in Northern area of J Province as research samples. However, in accordance with the purpose of Chinese criminal law and the policy of “education, reform and rescue” for juveniles, many illegal acts of juveniles have not entered the criminal trial procedure or have not been sentenced to imprisonment due to various subjective and objective circumstances. The number of juvenile offenders is small. Moreover, due to the differences in administrative divisions and the understanding and application of juvenile judicial policies in different regions, the number of juvenile offenders in custody in each province and city is also unevenly distributed. The same is true of J Province. At the same time, considering that once juvenile offenders enter the society, not only can they not get in touch with them, but also cannot clearly highlight the difference between juvenile offenders and ordinary juveniles, this research group selected juvenile offenders under control in the above cities and counties of J Province as the data source. Moreover, we chose incarcerated minors in relevant cities and counties of J Province as the survey objects, because their data samples can better reflect the difference between juvenile offenders and ordinary minors. As the result, we collected a total of 200 people’s data samples, including 41 in SZ, 30 in ZJ, 5 in TZ, 6 in HM and 38 in HZ. 10 in SY and 70 in XZ. After that, we excluded 9 data samples from 19-year-olds, 3 samples from 20-year-olds and 1 sample from 21-year-olds, and leaved a total of 187 data samples were taken as research objects.

In addition, this research topic also selected a group of ordinary teenagers as a comparative investigation object. This data came from the empirical study of the age growth of teenagers’ three capacity for criminal responsibility conducted by Professor Shang Y’s team of Shandong Normal University. After a preliminary overall selection and two supplementary selections, the study involved students from junior to senior three in S County, as well as some first-year students from S University, S Province, China, finally selecting a sample of 3,208 people (Shang et al., 2022). Then, we excluded 759 samples of students over the age of 18, remaining 2,449 samples for our research.

This research was approved and supported by the relevant departments from the cities and counties mentioned above, and the opinions of participants were solicited in advance for the issuance and filling of the questionnaire, among which all personal information related to the privacy of juveniles was hidden or not recorded. In order to comply with the policy requirements for the prevention and control of the novel coronavirus epidemic, researchers could not personally enter the juvenile offenders’ correctional center to issue questionnaires and fill out guidance. However, the questionnaire was contacted by prosecutors, issued by specialized personnel of the juvenile correction department and filled in with guidance throughout the process, which could guarantee the legitimacy and validity of the experimental data sources to a certain extent. According to the collected data, the basic situation of the sample is as follows, except for the two optional items of “crime committed” (missing 21) and “principal punishment declared” (missing 91).

### Measures

#### Brief-dialectical self scale (B-DSS)

In 2016, Spencer-Rodgers and colleagues developed a self-report questionnaire called the Dialectical Self Scale (DSS) (Spencer-Rodgers et al., 2004). The scale has been translated into many languages. We adopted the brief Chinese version (B-DSS), α = 0.71, with 14 items. The scale contains a 7-point scoring system from “very different” to “very much agree,” and encompasses the three dimensions of conflict tolerance, cognitive change, and behavioral change, thereby reflecting people’s dialectical thinking level. The higher the scale score, the higher the dialectical thinking level.

#### Self-control ability of middle school students questionnaire (SAMSSQ)

This questionnaire was developed by Wang Hongjiao and Lu Jiamei, scholars of the PRC. Adolescents’ capacity for self-control is mainly reflected in three dimensions: emotional self-control, behavioral self-control, and thinking self-control. The split-half reliability is 0.856 (Wang and Lu, 2004). The questionnaire has a total of 36 items, including 10 forward-scoring questions and 26 reverse-scoring questions. Each item uses a 5-point scoring system, ranging from “totally disagree” to “totally agree.” The higher the score, the stronger the self-control.

#### Basic empathy scale (BES)

There are many tools for measuring empathy, such as the widely used Interpersonal Response Indicator (IRI) in the PRC, but these scales have been questioned for confusing empathy with sympathy. Hence, for this study, we used the BES (Darrick & David, 2006). The BES is divided into two dimensions: emotional and cognitive empathy. The scale contains 20 items, including 8 items for negative scoring and 12 items for positive scoring; the higher the score, the greater the respondent’s empathy. Li et al. (2011) tested the structure of theoretical factors and the reliability and validity of the BES in the youth population of the PRC. They found that the BES met the relevant requirements of psychometrics (α = 0.777).

Demographic variables. We collected and coded the participants’ demographic information and filled in missing values. For grade variables (e.g., parents’ education level, family income), we used the average to fill in missing values. For disordered categorical variables (e.g., gender, family location), we used the mode to fill in missing values.

### Plan of analysis

We employed SPSS 23.0 to analyze the results. Before doing so, we calculated the scores of the B-DSS, SAMSSQ and BES (this score is the total score of each item on the scale). The missing values in the scores are filled in by the mean of the scores in the sample’s age group (Yu and Jin, 2015). After completing the above preparatory work. Firstly, the differences in B-DSS scores, self-control ability scores and BES scores between the sample data of 187 juvenile offenders and the sample data of 2,449 ordinary juveniles to be compared were investigated by independent sample *T* test. Secondly, one-way ANOVA was used to measure the impact of demographic variables on Dialectical thinking, self-control and empathy. Then the demographic variables were controlled as covariates to observe the development trend of dialectical thinking ability, self-control ability and empathy ability with age. Thirdly, using two-variable correlation analysis, we explored whether it was necessary to carry out multivariate analysis of variance (MANOVA) on each dimension of the B-DSS, SAMSSQ and BES.

## Results

This section is divided into two parts. The first part intends to present the differences between ordinary juveniles and juvenile offenders in capacity for dialectical thinking, self-control and empathy. Among them, the independent sample *T*-test for ordinary teenagers was based on part of the data collected by the Shang’s team mentioned above. The results for juvenile offenders are based on 187 sample data from Province J. Since the two studies adopted exactly the same standards of responsibility competence strategies, and the age and family conditions of the respondents were basically the same, the two could be compared at a certain level (see Table 1).

**Table 1:** Sample basic information (*N* = 187).

Basic information		*N*	Proportion (%)	Basic information		*N*	Proportion (%)
Gend-er	Male	177	95	Princip-al punish-ment pronou-nced*	Criminal detention	17	9.1
	Female	10	5		Not more than 1 year in prison	6	3.2
Age	13	1	0.5		1–2 years in prison	13	7.0
	14	2	1.1		2–3 years in prison	17	9.1
	15	22	11.8		More than 3 years in Prison	43	23.0	Vacancy	91	48.6
	17	64	34.2		Father’s educati-on level	No	11	5.9
	18	50	26.7	Primary		46	24.6	Secondary	119	63.6	College	3	1.6
	Hom-eland locati-on	Urban area	68	36.4		Bachelor	3	1.6
Rural area		119	63.6	Master or above		2	1.1
Hous-ehold inco-me	High	30	16.0	Other		3	1.6
	Middle	117	62.6	Mother’s educati-on level		No	17	9.1
	Low	40	21.4			Primary	59	31.6
Offen-ce comm-itted	Larceny	41	21.9		Secondary	100	53.5
	Robbery	8	4.3				
	Rape	69	48.1		College	3	1.6
	Intenti-onal injury	5	2.7		Bachelor	2	1.1
	Picking quarrels and provokin-g troubles	5	2.7		Master or above	1	0.5
	Other crimes	38	20.3		Other	5	2.7
	Vacanc-y	21	11.2				

^*^The so-called “Principal punishment” is a special term in the Criminal Law of the People’s Republic of China, including public surveillance, criminal detention, fixed-term imprisonment, life imprisonment and death penalty, among which criminal detention, fixed-term imprisonment and life imprisonment are all prison sentences.

### Independent sample *T* test

According to the data in Table 2, (1) In terms of the BES score, Levene test of homogeneity of the variance of the two populations is performed. In this example, the test statistic value *F* = 66.797 and its accompanying probability value *p* = 0.0.000, with a *p* value less than 0.01, indicate homogeneity of variances and the two population mean values are tested according to the data of equal variances. Thus, the test statistic value *t* = 19.849, the adjoint probability value *p* = 0.000, and the *p* value is less than 0.01, so the 95% confidence interval of the simultaneous difference is (7.739, 9.443), indicating that the difference between the two is extremely significant. (2) In terms of SAMSSQ score, the Levene test of homogeneity of two population variances. In this example, the statistical value *F* = 46.287 is accompanied by a probability value p = 0.000, and the *p* value is less than 0.05, indicating that the variances are not homogeneous, and the two population mean values are tested according to the data with unequal variances. Thus, the test statistic value *t* = 12.920, the adjoint probability value *p* = 0.000, and the p value is less than 0.01, so the 95% confidence interval of the difference at the same time is (12.105, 16.460), indicating that the difference between the two is extremely significant. (3) In terms of B-DSS score, according to Levene test, the test statistic value of this example is *F* = 0.123, its accompanying probability value *p* = 0.726, and the *p* value is greater than 0.05, indicating that the variances are homogeneous, and the two population means are tested according to the data of equal variances. Thus, the test statistic value *t* = 1.043, the adjoint probability value *p* = 0.297, and the p value is greater than 0.05: the 95% confidence interval of the difference at the same time is (−0.476, 1.559), indicating that the difference between the two is not statistically significant. Therefore, combined with the mean analysis of the two groups of data, the self-control ability of juvenile offenders (S _juvenile offenders_ = 109.30, S _ordinary juveniles_ = 123.59) and empathy ability (B _juvenile offenders_ = 63.86, B _ordinary juveniles_ = 72.45) were much lower than those of ordinary juveniles.

**Table 2:** Difference analysis of three kinds of abilities between ordinary juveniles and juvenile offenders.

Variables	ordinary juveniles		juvenile offenders		*t*
	*M*	SD	*M*	SD	
Dialectical thinking	61.29	6.820	60.75	7.093	1.04
Self-control	123.59	20.593	109.30	14.006	12.92^***^
Empathy	72.45	9.579	63.86	5.294	19.85^***^

^*^p < 0.05, ^**^p < 0.01.

The second part tries to present the relationship between age and the three abilities. Since the sample number of 13, 14 years old was very small to use (3 samples), the sample number utilized was 184 from juvenile offenders from 15 to 18 years old.

### Covariance analysis

Table 3 showed the difference analysis of demographic information, which mainly used independent sample T test and single factor ANOVA analysis. Independent sample T-test (including gender and family location) was used for binary variables, and univariate ANOVA analysis was used for three or more variables (including age, father’s educational level, mother’s educational level, and family income). According to the data in Table 3, except for the influence of mother’s educational level and family income on B-DSS score, the influence of other variables on B-DSS, self-control ability and BES score was not statistically significant.

**Table 3:** Difference analysis between demographic variables and the three ability scores.

Variable	B-DSS		SAMSSQ		BES	
	T/F	*p*	T/F	*p*	T/F	*p*
Age	0.467	0.857	0.965	0.458	0.610	0.747
Gender	1.004	0.317	−1.926	0.056	−0.007	0.994
Family location	1.299	0.196	−0.800	0.425	1.886	0.061
Father’s education level	1.909	0.071	0.723	0.653	1.172	0.321
Mother’s education level	3.293	0.003^**^	0.483	0.846	0.790	0.596
Family income	3.558	0.030^*^	0.564	0.570	1.532	0.219

^*^p < 0.05, ^**^p < 0.01.

Table 4 shows the scores of dialectical thinking ability, self-control ability and empathy ability at each age after controlling the variables of mother’s education level and family income. According to the results of covariance analysis, after controlling for maternal literacy and household income, the differences between age and B-DSS scores (*F* = 1.992, *p* = 0.226), SAMSSQ scores (*F* = 1.544, *p* = 0.205), and BES scores (*F* = 0.354, *p* = 0.786) were no longer statistically significant.

**Table 4:** Descriptive analysis of age variables and three ability scores.

Scale	B-DSS		SAMSSQ		BES	
Age	*M*	SD	*M*	SD	*M*	SD
15	**61.91**	**6.683**	**107.73**	**15.703**	**64.82**	**5.142**
16	**61.00**	**6.277**	**111.31**	**13.480**	**64.33**	**4.353**
17	**59.59**	**6.695**	**110.77**	**14.260**	**63.61**	**4.839**
18	**61.38**	**8.134**	**106.06**	**13.293**	**63.40**	**6.767**

B-DSS: the Brief-dialectical self scale, SAMSSQ: Self-control ability of the middle school students questionnaire, BES: Basic empathy scale, M: mean value, SD: standard value.

## Discussion

### Discussion of results

The research mentioned above analyzed the development of juvenile offenders’ dialectical thinking ability, control ability and empathy ability from horizontal and vertical perspectives, respectively, separately. Combined with the results of independent sample *T* test, the self-control ability and empathy ability of juvenile offenders were significantly different from that of ordinary juveniles, while the difference in dialectical thinking ability between juvenile offenders and ordinary juveniles was not statistically significant. According to the results of covariance analysis, except for mother’s education level and family income variables that had an impact on the score of B-DSS, other variables had no statistical significance on B-DSS, SAMSSQ and BES scores. In addition, there was no significant difference between age and B-DSS, SAMSSQ and BES scores.

#### Dialectical thinking ability

Dialectical thinking, as it’s called, is a form of mental organization presented as an open system model that “interacting with, changing, and potentially becoming integrated with each other over time, provided a greater degree of equilibrium than closed-system modeling taken by itself.” “This greater equilibrium could explain movements from formal to dialectical thinking in adults as they encountered limitations of or contradictions among closed system models, as well as offering a more adequate foundation for systematic inquiry” (Basseches, 1984). After controlling the variables of mother’s education level and family income, this study found that there was no significant difference between juvenile offenders’ dialectical thinking ability and that of ordinary minors at least between the ages of 15 and 18. In other words, juvenile offenders’ cognition and understanding of behavior deviance were at the same level as that of their peers as a whole. Then, a previous empirical study on middle school students showed that there were two important age turning points in adolescents’ dialectical thinking ability: it increased continuously before the age of 15, declined slightly between the ages of 15 and 18, and continued to increase after the age of 18 (Zhang, 2014). Although Zhang’s study mentioned that there is a downward trend in the dialectical thinking ability of middle school students between the ages of 15 and 18, and yet it did not mention that there is a significant difference in the dialectical thinking ability of middle school students between the ages of 15 and 18. Therefore, it could also be considered that this opinion indirectly supports the views of this paper.

#### Self-control ability

It has become a consensus that the significant increase in the risk of deviance and lawlessness is positively correlated with the immaturity of the self-control mechanism of juveniles during adolescence. A long-term follow-up survey of people who had participated in the delayed gratification task recovered that self-control has an individualized character, which reflects the importance of genes and the quality of social individuals’ resistance to stimuli in the development of high self-control (Casey and Caudle, 2013). Besides, studies have shown that parental behavior monitoring, parental self-control and adolescent self-control are significantly positively correlated. And there is a significant negative correlation between parental psychological monitoring and adolescents’ self-control ability (Deng et al., 2018). Therefore, the self-control ability of juvenile offenders is lower than that of their peers, which may be the result of individual, family and environmental factors.

#### Empathy ability

According to the horizontal and vertical observations, the BES scores of juvenile offenders not only differ greatly from those of ordinary adolescents, but also do not change with age. This is basically consistent with the dual processing model theory of empathic lifelong development, that is, the intensity of individual empathy ability remains relatively stable between adolescence and adulthood, and then gradually increases (Huang and Su, 2010). Empathy ability is the ability of juvenile offenders to accurately perceive and infer (empathic accuracy) the stable traits and attributes of others (Zaki and Ochsner, 2011; Hodges and Wise, 2016). The accuracy of empathy depends on the interaction of empathic object factors (willingness and ability to transmit information, channels to transmit information), empathic factors (information processing ability, willingness to empathize and immediate state), and social and cultural factors (Pan et al., 2022). Considering that an individual’s information processing capacity increases during adolescence (14–17 years) and peaks in early adulthood (25–35 years) (Richter et al., 2011), juvenile offenders should be no different from ordinary minors. The long-term neglect of empathic intention and the poor information transmission channel may be the important factors leading to their low empathic ability. In addition, it is considered that the brain regions related to emotion regulation and anxiety overlapped with the cognitive empathy system in the prefrontal cortex (Decety and Jackson, 2004). Glucocorticoids associated with anxiety can cross the blood–brain barrier and affect cognitive activities related to areas such as the amygdala, hippocampus, and prefrontal cortex (Lupien et al., 2007), and physiological factors of juvenile offenders may also play a role.

### Psychological reflection on juvenile delinquent’s ability of criminal responsibility

Although the criminal legislation of some countries insists on using the single standard of age of criminal responsibility to judge the responsibility capacity of minors, this standard is only an irrefutable presumption of responsibility capacity, rather than a confirmation of criminal responsibility capacity. In addition, in order to logically straighten out the relationship between the age system of criminal responsibility and the determination of criminal responsibility capacity, scholars in these countries began to seek for a substantial route to understand the criminal responsibility of juvenile offenders. They declared that the deviant behavior of a minor could also constitute a crime, except that those who did not reach the age of criminal responsibility were not subject to criminal punishment because of specific provisions of the criminal law. This study hoped to further focus people’s attention on the ability of criminal responsibility itself. In other words, the reason why juvenile criminals get “preferential criminal treatment” is not only because they are “less mature” than adults, but also because their ability of criminal responsibility is lower than that of their peers, and they cannot be expected to take full responsibility for this result. To be specific:

(1) Low self-control ability. As mentioned above, the self-control ability of juvenile delinquents in China is significantly lower than that of their peers, which is the result of the combined effects of their families, evil contacts and individuals themselves. However, these factors are not superimposed, but both influence each other and jointly cause this result. It may be arbitrary to completely deny the possibility of free choice for juvenile offenders, but it is unfair to ignore the influence of family and living environment on them. And even if there are three factors functioning at the same time, their effects are not necessarily in proportion. The self-control ability of parents and the monitoring behavior of children have a direct impact on the self-control ability of minors. According to the basic information of the sample, 63.6% of the respondents are from rural areas, and 4.3% of their fathers have a College education or above, and 3.2% of their mothers. Of course, we will not support the unfounded correlation between region, parental education level and children’s self-control ability. However, there are inevitably individuals who cannot develop self-control ability due to the lack of family discipline or unscientific discipline methods among these minors. At the same time, because of the homogeneity and consensus of social communication, they often make friends with peers who also lack such family discipline. Then, they develop traits and personalities that lack self-control and influence each other in long-term personality development. Similar phenomena have long been noted in the field of criminology in the United States (Adler et al., 2006).

(2) Low empathy ability. Similar to self-control ability, the lack of empathy ability of juvenile offenders can not ignore the role of family and environment. Some studies have shown that there is a significant negative correlation between childhood trauma and empathy (*r* = −0.076), and physical neglect (*r* = −0.095) and emotional neglect (*r* = −0.128) are both negatively correlated with empathy. In cognitive empathy, childhood trauma was significantly negatively correlated with perspective-taking (*r* = −0.127), but not with fantasy (*r* = −0.044). In affective empathy, childhood trauma was significantly negatively correlated with empathic attention (*r* = −0.148) and positively correlated with personal pain (*r* = 0.153) (Meng et al., 2019). I am afraid that most of these traumas are derived from acquired traumas in the family or living environment. In addition, from the perspective of experience, most of the empathic objects with minors are their family members, peers or elders with more contacts, and their long-term lack of empathy is inevitably significantly related to family and environment.

(3) The development of juvenile delinquent’s criminal responsibility capacity. Objectively speaking, although the dichotomous or multipartite of criminal age standard reflects the recognition of the difference between juvenile and adult offenders, it fail to reflect the difference in the criminal responsibility capacity of minors of different ages, because none of the three capacities showed a statistical difference with age. At the same time, the legislation is not consistent with the accepted idea of a State’s capacity to judge criminal responsibility. In order to reflect the difference between juvenile offenders and adult offenders, thus, only a minimum age of criminal responsibility standard can be created. For carrying out the difference between different juvenile offenders, thus, it is necessary to investigate the essence of criminal responsibility ability, so as to clarify the legitimacy of different individuals’ responsibility for specific crimes. Furthermore, since we have not effectively improved the criminal responsibility ability of juvenile offenders, we can hardly guarantee the effective suppression of the criminal motive, and we cannot focus merely on the protection of the rights of juvenile offenders. How to cultivate the social soil to promote juvenile delinquents to return to normal life is also the key through the joint efforts of the system among multiple subjects.

### Limitation and future studies

Although this study had drawn some conclusions in comparing the responsibility ability of ordinary juveniles and juvenile offenders, we would also face up to some limitations of this study.

Firstly, we conducted this study in the field in the PRC, and the development of juveniles varies greatly from country to country due to differences in history, culture, level of economic development and geography, so our conclusions cannot be universally applied to all nations. Each state should choose a minimum age of criminal responsibility according to the developmental situation of its juveniles and use the “malicious complementary age” as the configuration system to be applied.

Secondly, we used a questionnaire research method, which is somewhat subjective, and the relevant results depend heavily on how carefully the subjects filled out the questionnaire. In addition, there is a more objective, scientific research method—brain science research—which can be used in the future to explore the relationship between age and criminal responsibility more effectively.

Thirdly, there were limitations of subjects in this study. The small number of juvenile offenders in this study was due to the conservative approach that Chinese judicial authorities have to take due to concerns about the privacy and other rights of minors. The data of juvenile offenders in this study were sampled in Province J, and the data of ordinary minors were sampled in Province S. Although a large part of the data of juvenile offenders came from northern Jiangsu Province, this region was very close to S Province in geographical location, and they were connected in cultural origin for a long time, which can basically ensure the pertinence of the data comparison. However, after all, there were still part of the survey samples from southern Jiangsu and Central Jiangsu, and the follow-up study would further supplement the data of ordinary minors in this region to enrich the regional investigation of this comparative study. In addition, there are more Han residents in S and J provinces, so this research would not be with strong representative for sample of Yunnan, Xinjiang, Tibet and other areas inhabited by ethnic minorities. The PRC has a democratic centralized system, and the laws enacted by the National People’s Congress are valid nationwide. Therefore, subsequent studies should sample from populations nationwide to explore whether there are differences in dialectical thinking, self-control and empathy among adolescents from different regions and ethnic groups.

## Conclusion

There is no doubt that a great deal of effort has been made to address and reduce juvenile delinquency, but the impact of these efforts is still limited, especially in countries that are obsessed with the age of criminal responsibility. Nowadays, mankind has formulated a large number of criminal entities and procedural norms only to give juvenile offenders special criminal treatment. The purpose is nothing more than to let them feel the humanistic care of the state system through the tolerant treatment encountered in the criminal department, so that they will no longer breed or increase hatred against the legal order and the state system. Or, let them try to avoid so much social and cultural isolation that they lose the ability or willingness to consider themselves law-abiding and independent citizens. We highly affirms the value concept embodied in this approach, but also has to say that only we have opened half of the road. It’s suggested that the particularity of criminal treatment for juvenile should not only be reflected in the criminal punishment, but also in the criminal correction and assistant, that is, “both protection and correction.” The so-called “protection” is to protect the substantive and procedural rights and interests of minors to the maximum extent, to obtain favorable treatment for them in criminal proceedings according to law, and to create institutional conditions for their education and reform. The so-called “correction” is to strengthen the discipline and help of juvenile offenders, so as to promote their eventual recovery of physical and mental health, correction of character defects, cultivation of survival ability and return to normal life.

According to the specific circumstances of the crimes committed by juvenile offenders, there is no dispute about imposing different penalty measures and adopting different correction plans. If we affirm that juvenile delinquency comes from a series of distinct and complicated cause systems, the thinking on juvenile delinquency and social correction in this subject must also follow this idea. As far as juvenile offenders’ criminal treatment is concerned, the most realistic problem is how to give them preferential policies such as immunity from arrest, immunity from prosecution, acquittal, separate application of fine or suspension of execution within the normative framework. As far as the criminal punishment is concerned, because it mainly focuses on the discipline and punishment of juvenile offenders, it should be examined by the severity of their crimes and the possibility of their correction should be measured. Among them, the possibility of correction is based on the realistic possibility of improving the self-control ability and empathy ability of juvenile offenders, which has to consider the realistic reasons of juvenile offenders and the severity of subject reprehensibility. (a) When a minor commits or participates in the commission of an act that directly results in significant damage to the person, property or human dignity of another person, he or she shall be sentenced to substantial punishment, unless he or she is lured, deceived or coerced into committing the crime. (b) When a minor commits or participates in the commission of an act which has the consequence of causing serious damage to the person, property or human dignity of another person, the actual punishment shall be imposed on his or her reckless or deliberate subjective state, unless he or she is solely induced, deceived or coerced into committing the crime. (c) When a minor commits or participates in an act with slightly danger, he may not be sentenced to actual punishment, except for those who organize, instil or coerce others to commit it, and those who intentionally commit it repeatedly.

In the treatment of juvenile offenders, both the closed treatment in juvenile reformatory and the socialized treatment under community correction, emphasis should be placed on the strengthening of individual self-control ability and the recovery of empathy ability. In order to improve the self-control ability of juvenile offenders, no matter the closed correction in juvenile correctional centers or the socialized correction in community correction centers, the cultural skills or vocational skills training of minors should be strengthened. Most juvenile offenders are at or about to reach adolescence, and their pursuit of self-worth and sense of honor is no less than that of ordinary teenagers. Moreover, their world view has formed an early form, and it is difficult to change it by simply preaching, so we can only guide them to pursue self-realization methods acceptable to mainstream social concepts as much as possible, and make them emulate and internalize with relatively long-term individual incentives and group influence. In order to achieve this, an agreement can be reached between juvenile correctional centers and relevant enterprises based on the management experience of some places in China. During the period of correction, the former will organize juvenile offenders to contribute free labor to the design and production of the former’s products, while the latter will provide free literary and artistic skills, vocational and technical training for juvenile offenders who are subject to correction. And pay a certain remuneration to the former; In addition, juvenile offenders with outstanding performance can be given a series of institutional incentives such as family visits, meetings, and certificate awards by the former in accordance with regulations. On the other hand, in order to enhance the empathy ability of juvenile offenders, it is necessary to reshape and repair the relationship between juvenile offenders and family members, victims and the community, so as to reduce the probability of juvenile offenders joining illegal gangs again. This requires the joint efforts of juvenile correctional institutions, judicial organs, family organizations or groups, as well as schools and educational institutions. Juvenile correctional institutions or community correction institutions shall, on the premise of in-depth investigation of the basic situation of juvenile offenders, make suggestions to their family members through judicial organs, police organs, etc., and assist in promoting the restoration of the relationship between juvenile offenders and relevant subjects; If the guardian or close relatives of the minor offender are found to have bad behavior, they should be urged to correct it in time, and randomly follow up on the family information of the minor offender registered by the official.

## Data availability statement

The original contributions presented in the study are included in the article/Supplementary material, further inquiries can be directed to the corresponding author.

## Ethics statement

The studies involving humans were approved by the Academic Board of Shandong Normal University. The studies were conducted in accordance with the local legislation and institutional requirements. The participants provided their written informed consent to participate in this study. Written informed consent for participation in this study was provided by the participants' legal guardians/next of kin for minors under age 18.

## Author contributions

SL: Data curation, Methodology, Writing – original draft, Writing – review & editing. YS: Data curation, Writing – original draft, Writing – review & editing. FY: Data curation, Writing – original draft, Writing – review & editing. HL: Data curation, Writing – original draft, Writing – review & editing. DX: Data curation, Writing – review & editing. MZ: Data curation, Writing – review & editing. WJ: Data curation, Methodology, Writing – review & editing.
