# Supplementary material for: Peer assumption: an illusory consensus hidden in the criminal responsibility of juvenile offender—evidence from psychology
Source: Front Psychol. 2024 May 9;15:1321870. doi: 10.3389/fpsyg.2024.1321870 (PMC11113546; doi:10.3389/fpsyg.2024.1321870)
Supplement: Supplementary file 1 [file Data_Sheet_1.PDF]

Appendix A: LSD post tests for three abilities of the respondents of different ages

		Mean Deviation	Standard Deviation	P	95%CI	
**B-DSS**						
15	16	-0.21089	0.13917	0.131	-0.4855	0.0638
	17	-0.1743	0.13287	0.191	-0.4365	0.0879
	18	-0.43573**	0.13709	0.002	-0.7063	-0.1652
16	15	0.21089	0.13917	0.131	-0.0638	0.4855
	17	0.03659	0.10012	0.715	-0.161	0.2342
	18	-0.22484*	0.10566	0.035	-0.4333	-0.0163
17	15	0.1743	0.13287	0.191	-0.0879	0.4365
	16	-0.03659	0.10012	0.715	-0.2342	0.161
	18	-0.26143**	0.0972	0.008	-0.4532	-0.0696
18	15	0.43573**	0.13709	0.002	0.1652	0.7063
	16	0.22484*	0.10566	0.035	0.0163	0.4333
	17	0.26143**	0.0972	0.008	0.0696	0.4532
SAMSSQ						
15	16	0.25952	0.13868	0.063	-0.0142	0.5332
	17	0.15798	0.1324	0.234	-0.1033	0.4192
	18	-0.01073	0.13661	0.937	-0.2803	0.2588
16	15	-0.25952	0.13868	0.063	-0.5332	0.0142
	17	-0.10155	0.09976	0.310	-0.2984	0.0953
	18	-0.27025*	0.10528	0.011	-0.478	-0.0625
17	15	-0.15798	0.1324	0.234	-0.4192	0.1033
	16	0.10155	0.09976	0.310	-0.0953	0.2984
	18	-0.16871	0.09686	0.083	-0.3598	0.0224
18	15	0.01073	0.13661	0.937	-0.2588	0.2803
	16	0.27025*	0.10528	0.011	0.0625	0.478
	17	0.16871	0.09686	0.083	-0.0224	0.3598

**BES**						
15	16	0.00653	0.12684	0.959	-0.2438	0.2568
	17	0.00754	0.12109	0.950	-0.2314	0.2465
	18	-0.01378	0.12494	0.912	-0.2603	0.2328
16	15	-0.00653	0.12684	0.959	-0.2568	0.2438
	17	0.00101	0.09124	0.991	-0.179	0.1811
	18	-0.02032	0.09629	0.833	-0.2103	0.1697
17	15	-0.00754	0.12109	0.950	-0.2465	0.2314
	16	-0.00101	0.09124	0.991	-0.1811	0.179
	18	-0.02132	0.08858	0.810	-0.1961	0.1535
18	15	0.01378	0.12494	0.912	-0.2328	0.2603
	16	0.02032	0.09629	0.833	-0.1697	0.2103
	17	0.02132	0.08858	0.810	-0.1535	0.1961
